# Molecular detection of *Batrachochytrium dendrobatidis* (Chytridiomycota) and culturable skin bacteria associated with three critically endangered species of *Atelopus* (Anura: Bufonidae) in Ecuador

**DOI:** 10.7717/peerj.18317

**Published:** 2024-10-24

**Authors:** Jomira K. Yánez Galarza, Lenin Riascos-Flores, Leopoldo Naranjo-Briceño, Andrea Carrera-Gonzalez, H. Mauricio Ortega-Andrade

**Affiliations:** 1 Ingeniería en Biotecnología, Facultad de Ciencias de la Vida, Universidad Regional Amazónica Ikiam, Tena, Napo, Ecuador; 2 Grupo de Investigación en Biogeografía y Ecología Espacial (BioGeoE2), Universidad Regional Amazónica Ikiam, Tena, Napo, Ecuador; 3 Department of Animal Sciences and Aquatic Ecology, Faculty of Bioscience Engineering, Gent University, Gent, Belgium; 4 Research Institute for Nature and Forest, Brussels, Belgium; 5 Biotech Lab, Spora Biotech, Santiago, Región Metropolitana, Chile; 6 Herpetology Division, Instituto Nacional de Biodiversidad (INABIO), Quito, Pichincha, Ecuador

**Keywords:** Amphibia, Chytrid fungi, Central Andes Ecuador, Skin bacteria

## Abstract

Chytridiomycosis is a fungal disease responsible for massive amphibian die-offs worldwide, caused by the fungus *Batrachochytrium dendrobatidis* (Bd). Potential symbiotic relationships between frogs and the bacteria residing on their skin—referred to as skin-bacteria—may inhibit Bd growth, aiding in resistance to this lethal disease. This research had three main objectives: (1) to detect the presence of Bd in native populations of *Atelopus balios, A. bomolochos*, and *A. nanay* in the central Andes and coastal southern regions of Ecuador; (2) to identify the culturable skin-bacteria; and (3) to analyze differences among the bacterial communities in the three *Atelopus* species studied. Skin swabs were collected from two populations of *A. balios* (107–203 m a.s.l.) and one population each of *A. bomolochos* and *A. nanay* (3,064–3,800 m a.s.l.). These swabs served two purposes: first, to detect Bd using conventional PCR; and second, to isolate culturable bacteria, which were characterized through DNA sequencing, molecular phylogeny, and community composition similarity analysis (Jaccard index). Results showed that Bd was present in all species, with positive Bd PCR amplification found in 11 of the 12 sampled amphibians. The culturable skin-bacteria were classified into 10 genera: *Pseudomonas* (31.4%), *Stenotrophomonas* (14.3%), *Acinetobacter* (11.4%), *Serratia* (11.4%), *Aeromonas* (5.7%), *Brucella* (5.7%), *Klebsiella* (5.7%), *Microbacterium* (5.7%), *Rhodococcus* (5.7%), and *Lelliottia* (2.9%). The Jaccard index revealed that bacterial genera were least similar in *A. bomolochos* and *A. balios* (*J* = 0.10), while the highest similarity at the genus level was between *A. bomolochos* and *A. nanay* (*J* = 0.33). At the clade-species level, only *A. bomolochos* and *A. nanay* show common bacteria (*J* = 0.13). Culturable bacterial communities of specimens diagnosed as Bd positive (*n* = 10) or Bd negative (*n* = 1) share a J value of 0.1 at genus and 0.04 at species-clade level. The prevalence of Bd and the composition of cutaneous bacteria could be influenced by Bd reservoirs, *Atelopus* biology, and intrinsic environmental conditions. This research contributes to understanding the relationship between endangered Andean species and Bd, and explores the potential use of native skin-bacteria as biocontrol agents against Bd.

## Introduction

A lethal fungal panzootic has devastated the three orders of amphibians (Anura, Urodela, and Gymnophiona) worldwide, causing massive mortality events (Berger et al., 1998; Piotrowski, Annis & Longcore, 2004). This decline is primarily attributed to the fungus *Batrachochytrium dendrobatidis* (Bd) (Hanlon et al., 2018). Bd is the most recognized species related to the development of chytridiomycosis, a lethal disease affecting frogs, toads, and salamanders (Lips Karen, 2016).

Bd reproduces through asexual zoospores, which are equipped with a single flagellum that facilitates their movement in aquatic environments. Infection occurs when these motile zoospores contact skin of a susceptible host (Romero-Zambrano et al., 2021; Woodhams et al., 2018). Clinical signs of chytridiomycosis include lethargy, abnormal posturing, seizures, severe cutaneous disorders, and ultimately death (Marcum et al., 2010; Van Rooij et al., 2012). However, there is evidence that some amphibian species demonstrate greater tolerance to Bd than others, thereby avoiding the development of disease. This resistance has been linked to synergetic interaction of bacteria metabolites, antimicrobial peptides, and skin microbiota (Woodhams et al., 2014; Woodhams et al., 2007).

Like other organisms, amphibian skin hosts a layer of microorganism known as the cutaneous microbiota, which includes viruses, bacteria, and fungi (Bletz et al., 2013; Rosenthal et al., 2011; Woodhams et al., 2014). To understands the role of cutaneous microbiota in chytridiomycosis development, previous studies have shown that certain bacteria can produce secondary metabolites with the potential to either affect positively or negatively the host fitness (Liew et al., 2017; Morosini et al., 2006; Sun et al., 2023). For example, the molecules 2,4-diacetylphloroglucinol, produced by *Lysobacter gummosus* and indole-3-carboxaldehyde along with violacein produced by *Janthinobacterium lividum* demonstrated the potential to interfere with the growth of Bd (Niederle et al., 2019).

Historically, chemical treatment has been the primary method for the treatment of chytridiomycosis, yet there is a risk of adverse effect on the amphibian skin (Garner et al., 2009; Thumsová et al., 2024). In response, recent attention has shifted towards the potential of anti-Bd bacteria. This information is limited in Ecuador, with only one related study reporting the anti-Bd bacteria *J. lividum*, *Pseudomonas fluorescens* and *Serratia* sp., isolated from the high-land frog *Gastrotheca riobambae* (Bresciano et al., 2015). These bacteria have been described as a potential source of amphibian probiotics and Bd bio-controllers (Becker & Harris, 2010; Rebollar, Martínez-Ugalde & Orta, 2020). Furthermore, studies indicate that using autochthonous bacteria—naturally occurring in the amphibian’s native environment—can effectively prevent disease in the host species from which they are isolated. However, their effectiveness may not extend to other species (Bates et al., 2018; Becker et al., 2011; Harris et al., 2009; Rebollar, Martínez-Ugalde & Orta, 2020).

In Ecuador, the presence of Bd has been documented in eight out of the 24 provinces and in three out of the four regions (except for Galapagos). Bd has been identified in amphibian species within various families, including Bufonidae, Centrolenidae, Hylidae, Hemiphractidae, Craugastoridae and Leptodactylidae (Riascos-Flores et al., 2024). Notably, the Bufonidae family, which includes a significant number of threatened species, has experienced severe population declines that are potentially linked to Bd (Ortega-Andrade et al., 2021). Of these species, the genus *Atelopus* is highly threatened, with 25 species (44.6%) at risk (Ortega-Andrade et al., 2021).

This research had three aims: (1) to detect of the presence of Bd in native populations of *Atelopus balios, A. bomolochos*, and *A. nanay* in the central Andes and coastal southern regions of Ecuador; (2) to identify the culturable skin-bacteria; and (3) to determine the differences between the culturable bacterial communities characterized in the three *Atelopus* species studied. *A. nanay* is known to inhabit exclusively Cajas National Park (about 285 km^2^) in Azuay Province (IUCN, 2018), while the distribution of *A. balios* is confined to a threatened area of approximately 55 km^2^ (Pérez-Lara & Ramírez-Jaramillo, 2020). *A. bomolochos*, believed extinct until 2015, has to date been found only within the “Municipal conservation and sustainable use area” in the Cordillera Oriental of Azuay province (Ron, 2021; Ron & Merino, 2000). This study is expected to provide insights for the development of integrated conservation strategies, particularly in the realm of microbiota research and its role in amphibian health and disease resistance.

## Materials & Methods

### Study species and sampling sites

**Figure 1:**
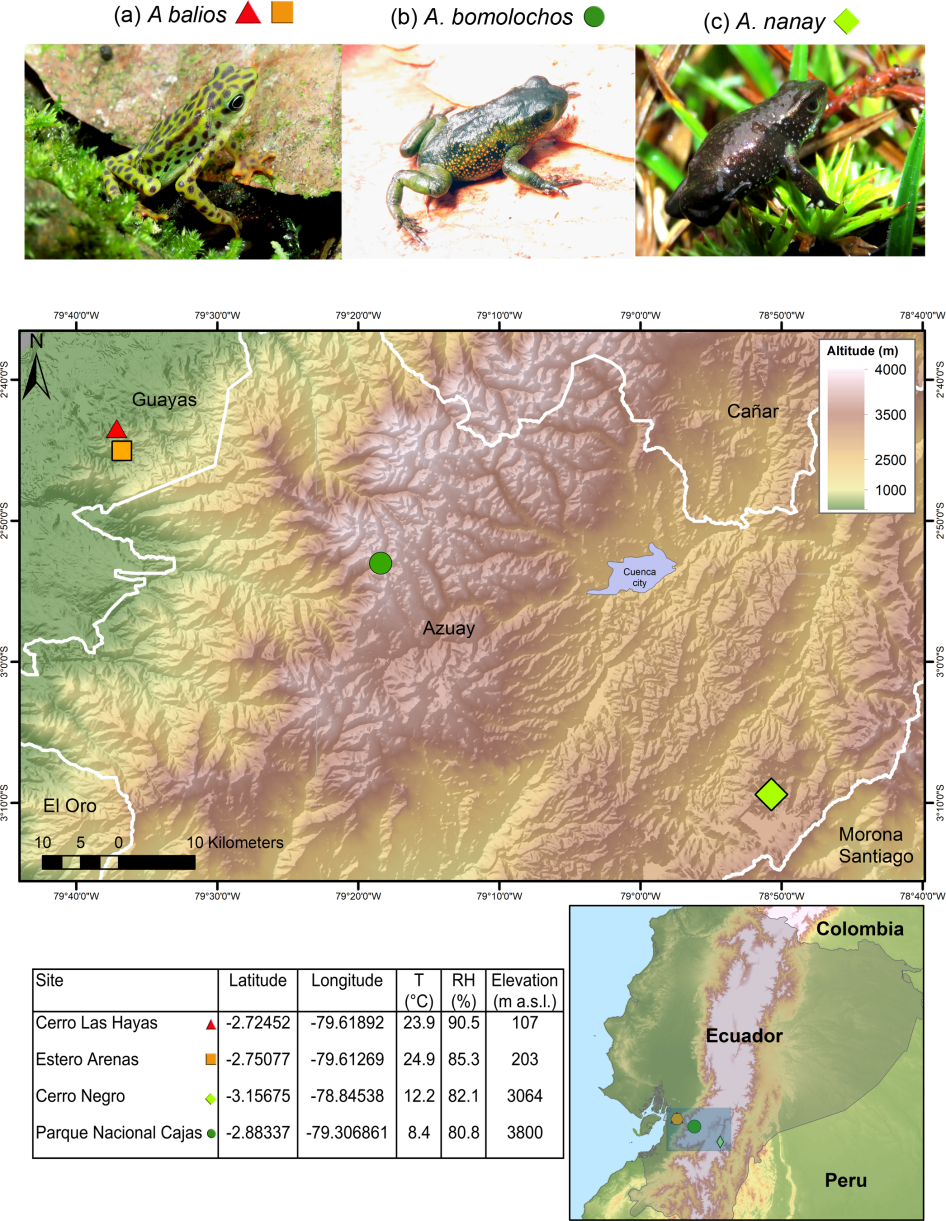
Map showing the studied locations in the highlands and coastal area of Ecuador. T, temperature. RH, relative humidity. m a.s.l., meters above sea level. Photographs by H. Mauricio Ortega-Andrade.

Data were collected as previously described in Yanez-Galarza (2022). The three *Atelopus* species are endemic from Ecuador and considered as critically endangered (CR) by the IUCN (Ortega-Andrade et al., 2021). Skin swabs were collected from two populations of the lowland inhabitant *A. balios* on Guayas province: “Cerro Las Hayas”(S02.72452, W79.61892) and “Estero Arenas” (S02.75077, W79.61269), in southwestern coastal Ecuador. Moreover, two highland inhabitant species were studied: *A. bomolochos* on “Cerro Negro” (S03.15675, W78.84538) and *A. nanay* in “Cajas National Park” (S02.88337, W79.30685), both located in Azuay province (Fig. 1). A field research permit (MAATE-DBI-CM-2021-0177) was obtained from the Ministry of Environment from Ecuador. Geographic data for the map in Fig. 1 were downloaded from SAVGIS http://www.savgis.org/ecuador.htm.

### Sampling strategy

Due to the limited number of adult specimens in the sampled areas (Cáceres-Andrade, 2014; Ortega-Andrade et al., 2021), amphibian sampling required a collaborative effort involving a team of four to seven people. Amphibians were located through visual encounter survey conducted along longitudinal transect surveys near permanent streams. To mitigate the risk of collecting transient bacteria, each specimen was captured using new disposable nitrile gloves and rinsed with sterilized ultrapure water (Lauer et al., 2008). The swabbing procedure was performed in accordance with the protocols established by Angulo et al. (2006). Duplicates of CITOSWAB Series® and a microbiological collection and transport system (Amies Charcoal gel swabs) were used for each specimen. Control samples were obtained by exposing a sterile swab to the open air at the study sites for 5 s to capture environmental microbes present in the surrounding air. The first swab of each duplicate and the control swabs were immediately stored in their transport system according to manufacturer’s specifications and stored at −4 °C for bacterial isolation. The second swab was preserved in a 1.5 mL cryovial containing 400 µL of lysis buffer (Tris-HCl 0.18 M; EDTA 10 mM, SDS 1%, pH 8.2), refrigerated at −4 °C and processed for DNA isolation within 24 h after sampling in a field lab (Riascos-Flores et al., 2024). In this process, samples used for DNA extraction and subsequent PCR reaction for the presence of Bd were also considered negatives controls for this study. After sampling, specimens were released at the same place of capture. No specimens were euthanized.

### Bacterial isolation

Each swab was plated using the streaking method on Luria-Bertani (LB) agar (37 g/L) in duplicate and incubated at 30 °C for 48 h. Bacterial morphotypes were defined according to the macroscopic characteristics of the obtained colonies (*i.e.,* color and form). Single colonies of each bacterial morphotype per sample were streaked on fresh LB agar to obtain axenic cultures. Each isolate was cryopreserved in Mueller Hinton broth (21 g/L) with 30% glycerol at −80 °C.

#### DNA isolation and PCR amplification

DNA extraction for Bd detection was performed according to the protocol described by Riascos-Flores et al. (2024). PCR amplification was performed using a miniPCR™ mini16 thermal cycler. The specific primers ITS1-3 Chytr-F (5′-CCTTGATATAATACAGTGTG CCATATGTC-3′) and 5.8S Chytr-R (5′-AGCCAAGAGATCCGTTGTCAA-3′) (Boyle et al., 2004) were used, resulting in a 146 bp amplicon. Reactions contained 7.5 µL of autoclaved MilliQ H_2_O, 1 µL of each primer at a concentration of 10 mM, 12.5 µL of TaqMan Environmental Master Mix 2.0, and 3 µL of DNA for a total volume of 25 µL per reaction. The thermal cycle was programmed in the miniPCR App v2.0 software with the following conditions: initial denaturation at 95 °C for 120 s, 35 cycles of denaturation at 95 °C for 60 s, annealing at 60 °C for 30 s and extension at 72 °C for 30 s, and final extension at 72 °C for 300 s.

#### Molecular identification of bacteria

Bacterial DNA isolation was performed following the protocol for isolating genomic DNA from gram-positive and gram-negative bacteria of the modified Promega Wizard® genomic DNA purification kit. PCR amplification was performed using a miniPCR ™ mini16 thermal cycler. The 16S bacterial rRNA of each bacterial morphotype isolated from each sample was amplified using the primers 16s-F (5′-GGAGGCAGCAGTAGGGAATA-3′) and 16s-R (5′-TGACGGGCGGTGAGTACAAG-3′) (Persson & Olsen, 2005). The PCR master mix contained 15.5 µL of sterile MilliQ H_2_O, 2.5 µL of Invitrogen’s 10X Buffer Green, 0.75 µL of MgCl_2_, 0.5 µL of dNTPs, 0.5 µL of each primer (10 µM), 0.1 µL of Invitrogen’s Platinum Taq DNA Polymerase, and 5 µL of DNA (25 ng/µL) for a final volume of 25 µL per reaction. PCR conditions were as follows: initial denaturation at 95 °C for 300 s, 34 cycles of denaturation at 94 °C for 60 s, annealing at 54 °C for 45 s and extension at 70 °C for 60 s, followed by a final extension at 70 °C for 480 s.

PCR products were visualized by electrophoresis in a blueGel ™ electrophoresis with built-in transilluminator equipment, using 2% agarose gel in TBE 1X with 1X GelGreen ™ nucleic acid stain. The 100 bp DNA ladder (Promega, Madison, WI, USA) was used to confirm the size of the amplified products. Bd (146 bp) and bacteria (1,062 bp). Amplicons were purified from agarose gel using the Wizard® SV Gel and PCR Clean-Up System from Promega.

### Sequencing data processing

For Bd detection the purified products were sent to Macrogen Co. Ltd. (South Korea) for Sanger DNA sequencing. Sequences were trimmed, edited, and assembled using Geneious v.5.4.7 software. For 16S rRNA sequences were identified using BLAST/n against the complete GenBank nucleotide database with default parameter settings (Flechas et al., 2012).

### Taxonomy and phylogenetic analysis of the 16S rRNA gene

The 16S rRNA consensus sequences obtained and additional 135 sequences (>1,000 bp) from bacteria from Genbank were aligned using Geneious v.5.4.7. Additionally, the *Synechococcus elongatus* sequence (AB871649) was used as an outgroup.

Mesquite v3.0 was used to export the aligned matrix in NEXUS format for MrBayes with the default parameterization. jModelTest2 software was used to test the best nucleotide substitution model on the CIPRES platform https://www.phylo.org/.

Once the best nucleotide substitution model was stablished, the phylogenetic analysis was performed with Bayesian methods on the aligned matrix in MrBayes v3.2.2 in CIPRES. The following parameters were configured: two parallel sections of the Metropolis coupled Monte Carlo Markov chain, two independent runs, 20 million generations, with three hot chains (temperature 0.2), saving a tree and its statistics every 1,000 generations; and a burn fraction of 25% of the trees.

Tracer v1.7.1 software was used to validate the phylogenetic models based on the distribution pattern and stability of the likelihood values evaluated from the Effective Sample Size (ESS > 200) parameter estimates across generations.

The taxonomic assignation was based on the Basic Local Alignment Search Tool (BLAST/n) similarity and the phylogenetic position of each sample to bacterial lineages. Unconfirmed genetic samples were labeled with “*affinis*” (aff.) or *“confer*” (cf.) to refer to similar or comparative taxonomic identities. Samples that formed a cluster with a probable species and showed a support value ≥ 0.7 and <0.9 were labelled as “*affinis*” (aff.). Samples that either formed a cluster with a probable species and showed a support value <0.7, or clustered alone but closely with a clade suggesting a close genetic relationship, were labeled as *“confer*” (cf.).

### Community composition

A similarity analysis was performed to compare the composition of culturable bacterial communities among species and between positive and negative samples with the Jaccard coefficient (J) in PAST software (Hammer et al., 2001).

## Results

### Bd detection in native populations

A total of 12 specimens sampled from *Atelopus* toads were swabbed. Bd was detected across all four sampling sites and in all three *Atelopus* species, showing a prevalence of 91.7% (11 out of 12). Specifically, positive BD was found in two individuals of *A. nanay* (HMOA 2397, HMOA 2390) from Cajas National Park, two *A. bomolochos* from Cerro Negro, and seven *A. balios*—five from Cerro Las Hayas (HMOA 2415-2419) and two from Estero Arenas (HMOA 2420-42421). No signs of chytridiomycosis were observed in any of the specimens, including the deceased individual identified as HMOA 2399 of *A. nanay*, which tested positive for Bd. This specimen was found dead beneath a rock in a stream at Cajas National Park. Only one *A. nanay* specimen, HMOA 2398, tested negative for Bd. Additionally, no amplification was detected in the negative control swabs from each location.

### Bacterial taxonomy and phylogeny identification of culturable skin-bacteria

Identification of 16S rRNA from a total of 35 bacteria morphotypes through BLAST/n search yielded 11 genera (*Acinetobacter*, *Aeromonas*, *Brucella*, *Klebsiella*, *Lelliottia*, *Microbacterium*, *Pantoea*, *Pseudomonas*, *Rhodococcus*, *Serratia,* and *Stenotrophomonas*) belonging to 10 families, 6 orders, 3 classes, and 2 phyla (Table S2). However, maximum scores, percentage of query coverage, and percentage identity of genetic samples coincided with multiple sequences from different species. One hundred seventy sequences were downloaded from GenBank to recover the phylogeny, based on BLAST/n similitude for the 16S rRNA gene.

**Figure 2:**
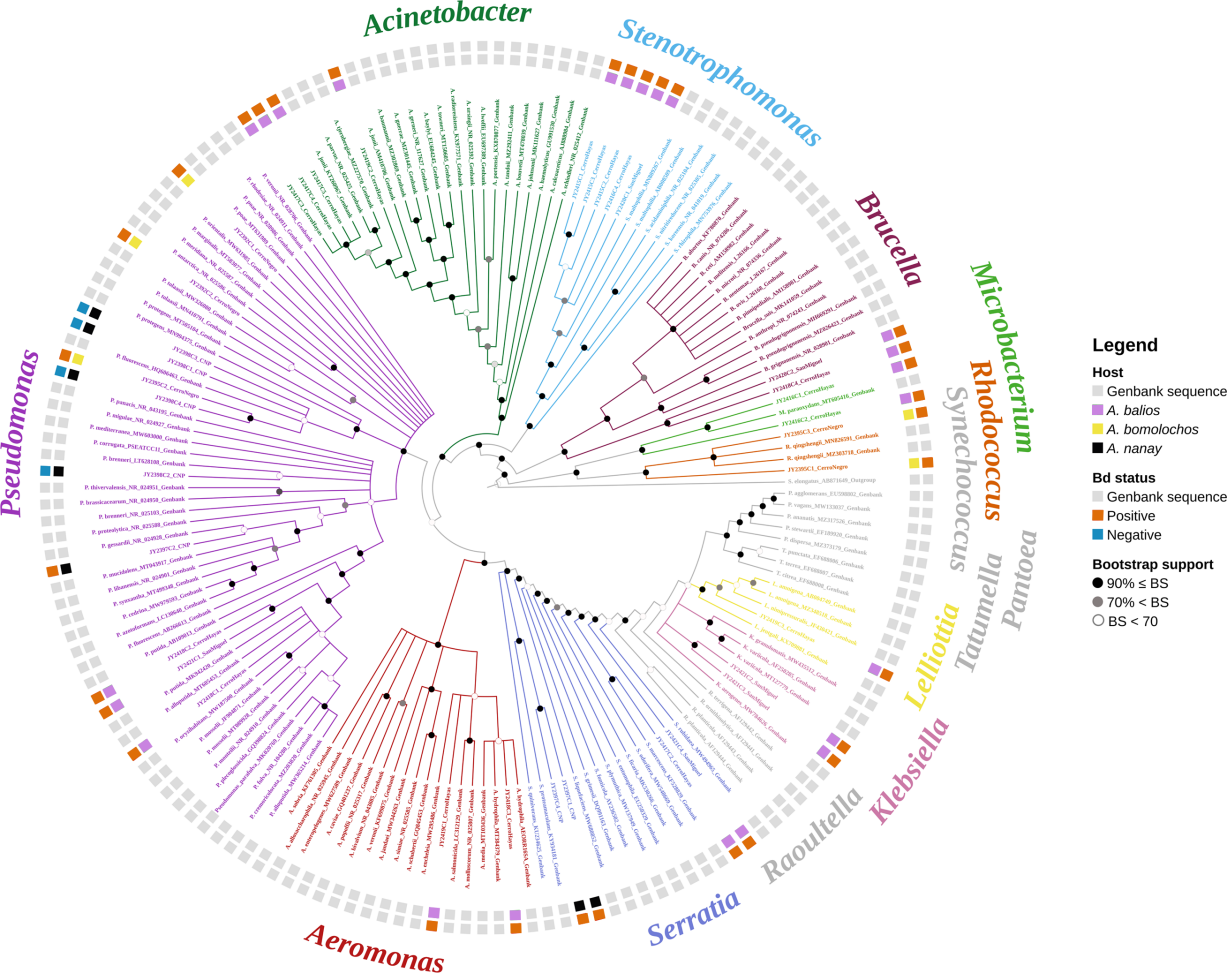
Optimal maximum likelihood tree from the 16s rRNA gene of a matrix (log-likelihood = −16777.01; 1349 aligned sites) showing the phylogenetic relationships of 35 bacteria isolates sequences joined to 135 GenBank sequences. The color per clade label indicates genera. The two rings in the outer circle correspond to the character state: the host species from which the bacteria were isolated (inner ring), and the host species Bd negative/positive status (outer ring). Supporting values of non-parametric bootstrap (colors on the nodes) are shown.

**Figure 3:**
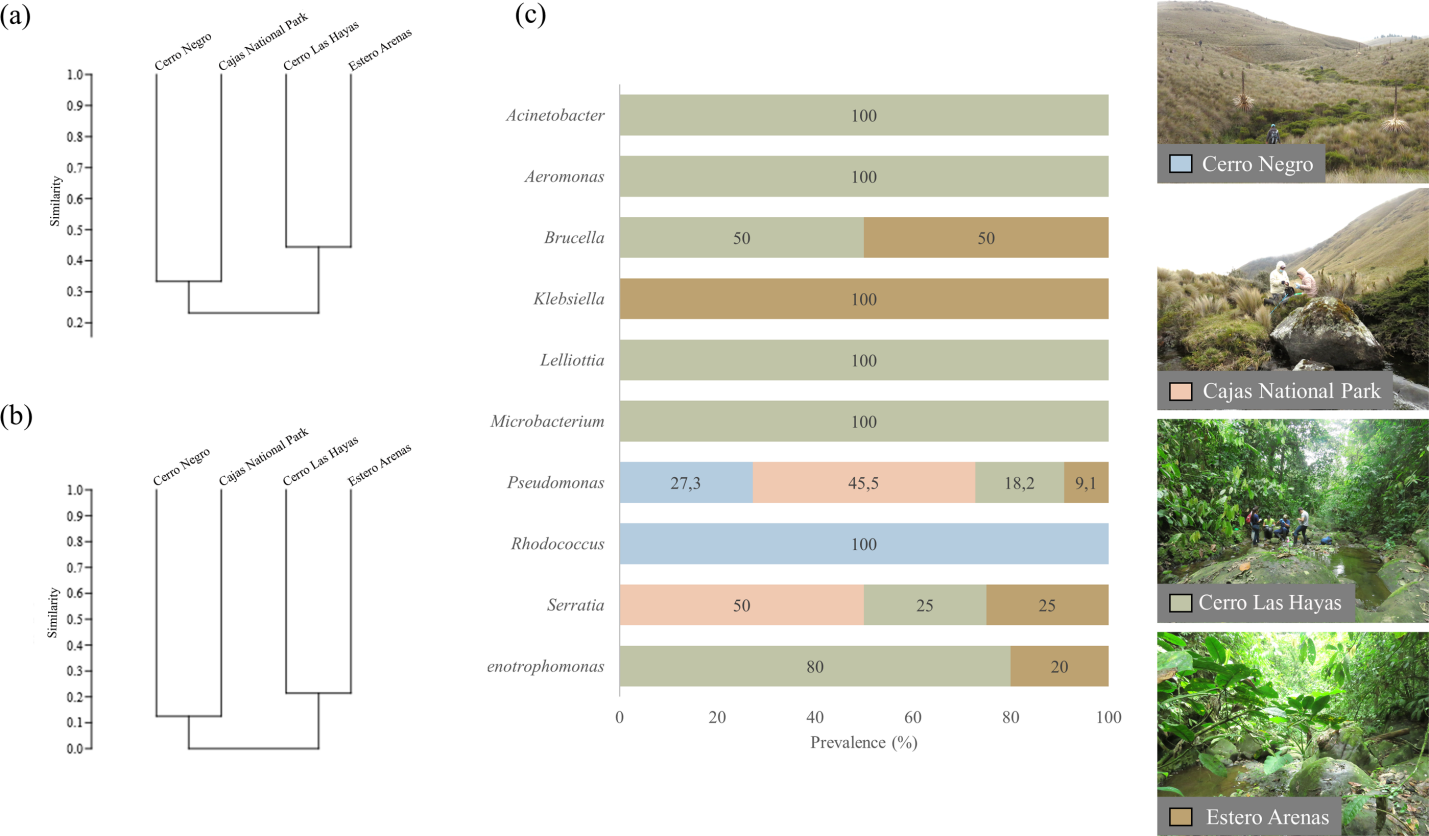
UPGMA dendrograms showing the relationship between bacterial communities and four sampling sites. (A) Genera and (B) clade-species level, based on Jaccard Similarity index; (C) genera prevalence by sampling site. Photographs by H. Mauricio Ortega-Andrade.

The best topology (log-likelihood-16777.01) was obtained from a matrix with an extended set of 1349 characters (Fig. 2, Fig. S1). It allowed to identify 22 clades belonging to 10 genera: *Pseudomonas* (31.4%), *Stenotrophomonas* (14.3%), *Acinetobacter* (11.4%), *Serratia* (11.4%), *Aeromonas* (5.7%), *Brucella* (5.7%), *Klebsiella* (5.7%), *Microbacterium* (5.7%), *Rhodococcus* (5.7%), and *Lelliottia* (2.9%) (Fig. 3 and Table S2). *Pantoea* was renamed as *Klebsiella* based on the phylogenetic position (Fig. 2). No attachment of bacterial DNA sequences from this study to BLAST/n suggested species was observed, and Bayesian posterior probabilities <0.7 were also noted. Hence, one (2.9%) of the samples were assigned as *affinis*, whereas 28 (80%) were assigned as *confer*.

### Comparison between culturable bacteria communities

**Figure 4:**
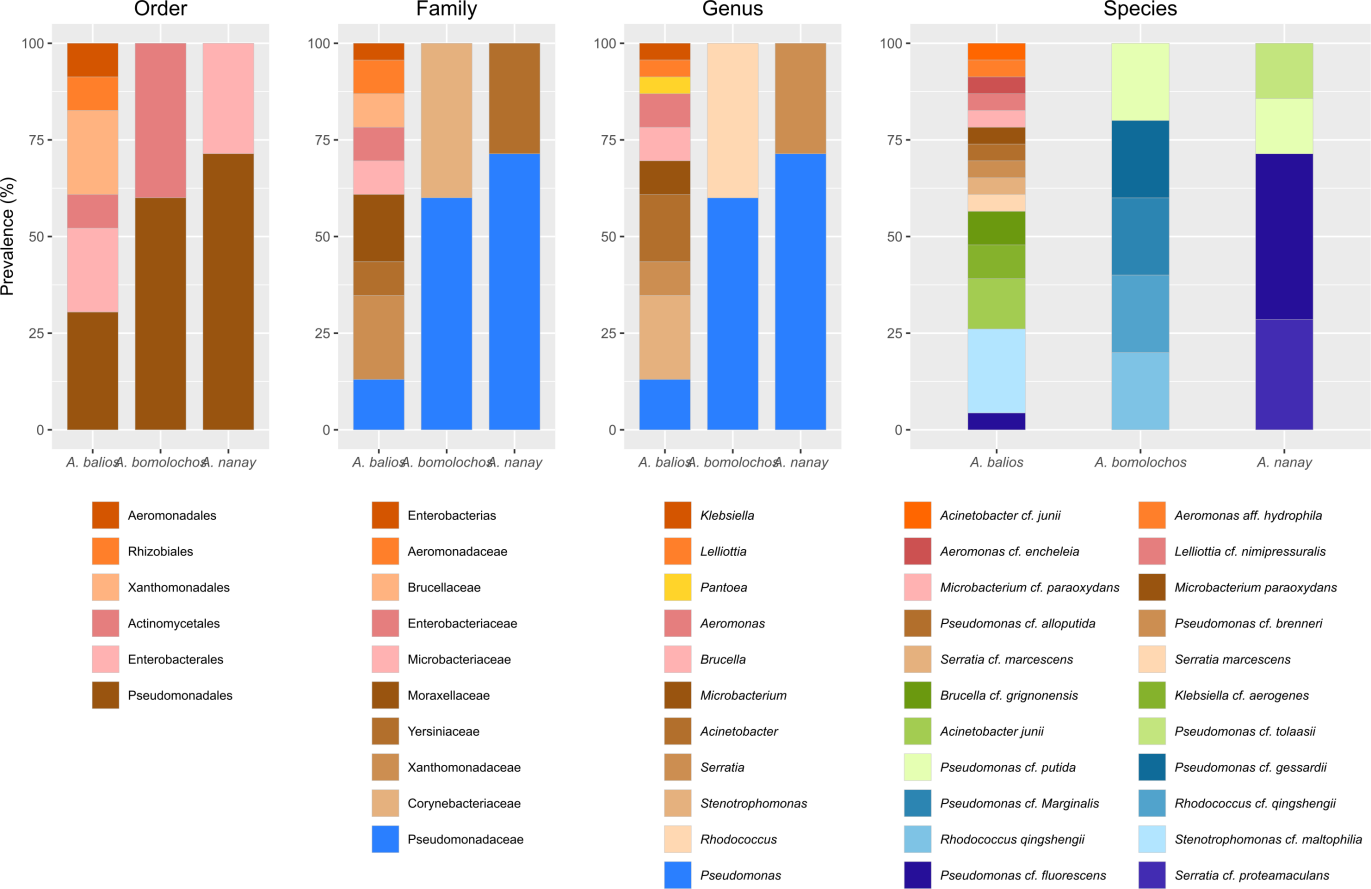
*Prevalence of bacteria* isolated from *Atelopus* toads. Cutaneous microbiota associated to sampled *A. balios*, *A. bomolochos*, and *A. nanay* toads sorted by order, family, genus, and species level.

Clustering by the UPGMA hierarchical method (see Fig. 4, section a and b) show two major clusters: one composed by the culturable bacterial communities from the highlands and the other from the lowlands. This indicates that bacterial communities are grouped according to their respective environments (Fig. 4). Results from the Jaccard similarity coefficient (J) show that bacterial genera were least similar in *A. bomolochos* and *A. balios* (*J* = 0.10), while the highest similarity at the genus level was between *A. bomolochos* and *A. nanay* (*J* = 0.33). At the clade-species level, only *A. bomolochos* and *A. nanay* show common bacteria, with a Jaccard similarity of *J* = 0.13 (Table S3).

Culturable bacterial communities of specimens diagnosed as Bd positive (*n* = 10) or Bd negative (*n* = 1) share a J value of 0.1 at genus and 0.04 at species-clade level. The genera *Pseudomonas* was the only genus found in both infected and uninfected individuals. In contrast, *Acinetobacter*, *Aeromonas*, *Brucella*, *Klebsiella*, *Lelliottia*, *Microbacterium*, *Rhodococcus, Serratia* and *Stenotrophomonas* were only found in infected toads (Fig. 4).

## Discussion

### Detection of Bd in native species

Bd presence and skin culturable bacteria communities were analyzed in native populations of three critically endangered *Atelopus* species in Central Andes and coastal Ecuador. The decline of *A. bomolochos* has been linked to Bd infection, evidenced by the histological diagnosis of a museum specimen collected in 1980 (Ron & Merino, 2000). Since its rediscovery in 2015, there have been no reports of Bd with this population. However, our study shows that specimens from Cerro Negro are infected by Bd, representing another threat to this population, which already faces habitat alteration (Siavichay, 2018).

For the *A. nanay* population in the Cajas National Park, this study represents the second report of Bd in individuals from the area (Cáceres-Andrade, 2014). Moreover, the detection of Bd in one individual found dead, highlights ongoing risk but does not conclusively attribute death directly related to chytriomycosis. The persistence of Bd in Cajas National Park can be attributed to a “latent” stage of the fungus, which enables survival outside of hosts (Mitchell et al., 2008) as well as suitable biotic and/or abiotic conditions for its development (Lambertini et al., 2021). Non-declining amphibian species (*i.e., Gastrotheca* spp. and *Pristimantis* spp.) may act as Bd reservoirs (Hudson et al., 2019). Cool temperatures at high tropical elevations, such as in Cajas National Park and Cerro Negro have been described to favor the growth of Bd (Lambertini et al., 2021; Piotrowski, Annis & Longcore, 2017).

This study represents the first report of Bd infection in *A. balios*, a lowland rainforest inhabitant species, in Guayas province. This represents the lowest elevation site for Bd presence reported in Ecuador, at 107 m above sea level. Additionally, a previous Bd detection in the amphibian population was recorded 35.7 km from our sampling site, at an elevation of 350 m above sea level (IUCN SSC Amphibian Specialist Group, 2018; Pérez-Lara & Ramírez-Jaramillo, 2020).

### Differences in cultivable microbiota

As expected, culturable bacterial communities diverged between *Atelopus* species. Despite belong to the same genus, the sampled species exhibited distinct environmental and biological characteristics including host immunity, skin toxin production and frequency of skin shedding. These characteristics are likely to have influenced the composition of their microbiota, which was observed to vary across species. This study is consistent with previous evidence that amphibian skin bacterial communities tend to be host species-specific (Kruger, 2020; McKenzie et al., 2012b; Solomon et al., 2017; Walke et al., 2017). The higher similarity value (J) between *A. bomolochos* and *A. nanay* may be linked to their biogeography, which is restricted to highland forests and paramos in the central Andes of Ecuador. In contrast, *A. balios* is distributed in a completely different environment, in tropical conditions. Other studies have indicated that tropical habitats provide optimal conditions for bacterial diversity and richness (Bresciano et al., 2015; Nottingham et al., 2018).

Previous studies described the isolation of *Acinetobacter, Aeromonas, Brucella*, *Microbacterium*, *Klebsiella*, *Pseudomonas*, *Rhodococcus, Serratia* and *Stenotrophomonas* from the skin of different hosts (Barra, Simmaco & Boman, 1998; Bates et al., 2018; Becker et al., 2021; Bresciano et al., 2015; Catenazzi et al., 2018; Flechas et al., 2017; Kanchan et al., 2021; Khalifa, AlMalki & Bekhet, 2021; Latheef et al., 2020a). To date, there have been no reports of *Lelliottia* isolations from amphibian skin. Of the ten genera identified, two (*Pseudomonas* and *Rhodococcus*) were present in *A. bomolochos*, two(*Pseudomonas* and *Serratia*) in *A. nanay*, and all ten, except *Rhodococcus,* in *A. balios*. This last finding can be compared with other lowland toads such as *Atelopus aff. limosus*, *A. spurrelli*, and *A. elegans*. Flechas et al. (2012) identified eight genera of culturable skin bacteria. These were isolated from *A. elegans* (*n* = 82), five from *A. aff. limosus* (*n* = 80)*,* and six from *A. spurrelli* (*n* = 78). Three of these bacteria are shared with *A. balios* (*Acinetobacter*, *Pseudomonas*, *Stenotrophomonas*). Subsequently, Flechas et al. (2017) identified 22 genera distributed among the same *Atelopus* species in the wild, with 19 genera isolated from *A. elegans* (*n* = 5), four from *A. aff. limosus* (*n* = 8), and five from *A. spurrelli* (*n* = 5). Six of the bacteria were found to be shared with *A. balios*, namely *Pseudomonas*, *Acinetobacter, Stenotrophomonas, Microbacterium*, *Klebsiella* and *Aeromonas*.

### The effect of Bd on skin microbiota composition remains debated

Some studies have found no variation in the skin microbiota caused by Bd (Belden et al., 2015; Kruger, 2020), while others have reported changes in the function and structure of the skin microbiota (Becker et al., 2015; Jani & Briggs, 2014; Walke et al., 2015). Our study results indicate that individuals infected with Bd and those uninfected host distinct culturable bacterial communities. However, due to the limited number of individuals evaluated, we recommend conducting a more extensive sampling campaign and analysis.

Although we could not confirm the bacterial strains at the species level, some isolates belong to genera known for their anti-Bd properties, such as *Acinetobacter, Pseudomonas, Serratia*, and *Stenotrophomonas* (Bresciano et al., 2015). However, we did not find *Janthinobacterium* in our samples (Niederle et al., 2019). Among the probable species found is *Serratia marcescens*, known to produce a potent antifungal metabolite. This bacterium had shown up to 100% inhibition against Bd (Becker et al., 2021). Other potential bacteria who have demonstrate its capability to inhibit Bd and modulate the host’s ability to survive to chytridiomycosis are *Serratia marcescens*, and *Pseudomonas* (*e.g.*, *P. entomophila*, *P. azotoformans*, *P. fluorescens*) (Catenazzi et al., 2018; Robak & Richards-Zawacki, 2018). In contrast, some bacteria identified are known amphibian pathogens, such as *Brucella* and *Aeromonas hydrophila*. *Brucella* has been reported in amphibians, with demonstrated zoonotic potential (Rouzic et al., 2021). Other genera may play a synergetic role with Bd within the skin microbiota. For example, *Microbacterium* has been shown to produce nutritive compounds, which promotes the Bd growth (Becker et al., 2015). On the other hand, *Aeromonas hydrophila* is well known for causing ‘red leg’ disease (Densmore & Earl Green, 2007), and has recently associated with severe pathological clinical signs in eggs and adult frog individuals (Khalifa, AlMalki & Bekhet, 2021).

## Conclusions

This study represents the first comprehensive investigation of the skin microbiota of critically endangered *Atelopus* species in Ecuador. The results revealed a prevalence of Bd infection across these species in areas known as the last refuges for these critically endangered populations. These findings highlight the urgent need for conservation efforts to better understand this zoonotic disease and generate valuable information for conservation strategies.

A total of ten bacterial genera were identified from the skin of the *Atelopus* species, including *Pseudomonas*, which has previously been noted for its potential to inhibit Bd infections. Conversely, other identified bacteria revealed the presence of potential emerging pathogens. Overall, this study highlights the need for further research involving additional individuals from the region, focusing on different populations and other species.

##  Supplemental Information

10.7717/peerj.18317/supp-1 Figure S11 Expanded optimal maximum likelihood tree from the 16s rRNA gene of a matrix (log-likelihood = -16777.01; 1349 aligned sites) showing the phylogenetic relationships of 35 bacteria isolates sequences joined to 135 GenBank sequences Section (a) of the phylogeny.

10.7717/peerj.18317/supp-2 Figure S12 Expanded optimal maximum likelihood tree from the 16s rRNA gene of a matrix (log-likelihood = -16777.01; 1349 aligned sites) showing the phylogenetic relationships of 35 bacteria isolates sequences joined to 135 GenBank sequences Section (b) of the phylogeny

10.7717/peerj.18317/supp-3 Figure S13 Expanded optimal maximum likelihood tree from the 16s rRNA gene of a matrix (log-likelihood = -16777.01; 1349 aligned sites) showing the phylogenetic relationships of 35 bacteria isolates sequences joined to 135 GenBank sequences Section (c) of the phylogeny

10.7717/peerj.18317/supp-4 Table S1 Preliminary information of the bacterial isolates was given by BLASTn analysis Data on isolated strains, taxonomic classification and maximum identity from BLASTn

10.7717/peerj.18317/supp-5 Table S2 Summarized similarity measures of the identified bacterial species using 16S rRNA gene by BLASTn analysis Isolated code, probable species, scores and accession Genbank accession numbers

10.7717/peerj.18317/supp-6 Table S3 Bacterial identification by phylogenetic analysis Distance percentages between the sequences of the isolated strains, and the DNA sequences of the possible species from GenBank.

10.7717/peerj.18317/supp-7 Supplemental Information 7 Access number to Genbank, taxonomy identity, strain codes, hosts and locations of bacterias Each strain includes taxonomic, host and geographic metadata and access numbers to Genbank

## Additional Information and Declarations

### Field Study Permissions

The following information was supplied relating to field study approvals (*i.e.*, approving body and any reference numbers): The Ministerio de Ambiente from Ecuador provided research permit number MAATE-DBI-CM-2021-0177

### DNA Deposition

The following information was supplied regarding the deposition of DNA sequences: The sequences are available at GenBank: OM859075.1–OM859109.1.

### Data Availability

The following information was supplied regarding data availability: The raw data of bacteria strains, accession numbers to GenBank, hosts and locations are available in the Supplementary Files.
