# Supplementary figures and images for: Molecular detection of Batrachochytrium dendrobatidis (Chytridiomycota) and culturable skin bacteria associated with three critically endangered species of Atelopus (Anura: Bufonidae) in Ecuador
Source: PeerJ. 2024 Oct 24;12:e18317. doi: 10.7717/peerj.18317 (PMC11512805; doi:10.7717/peerj.18317)

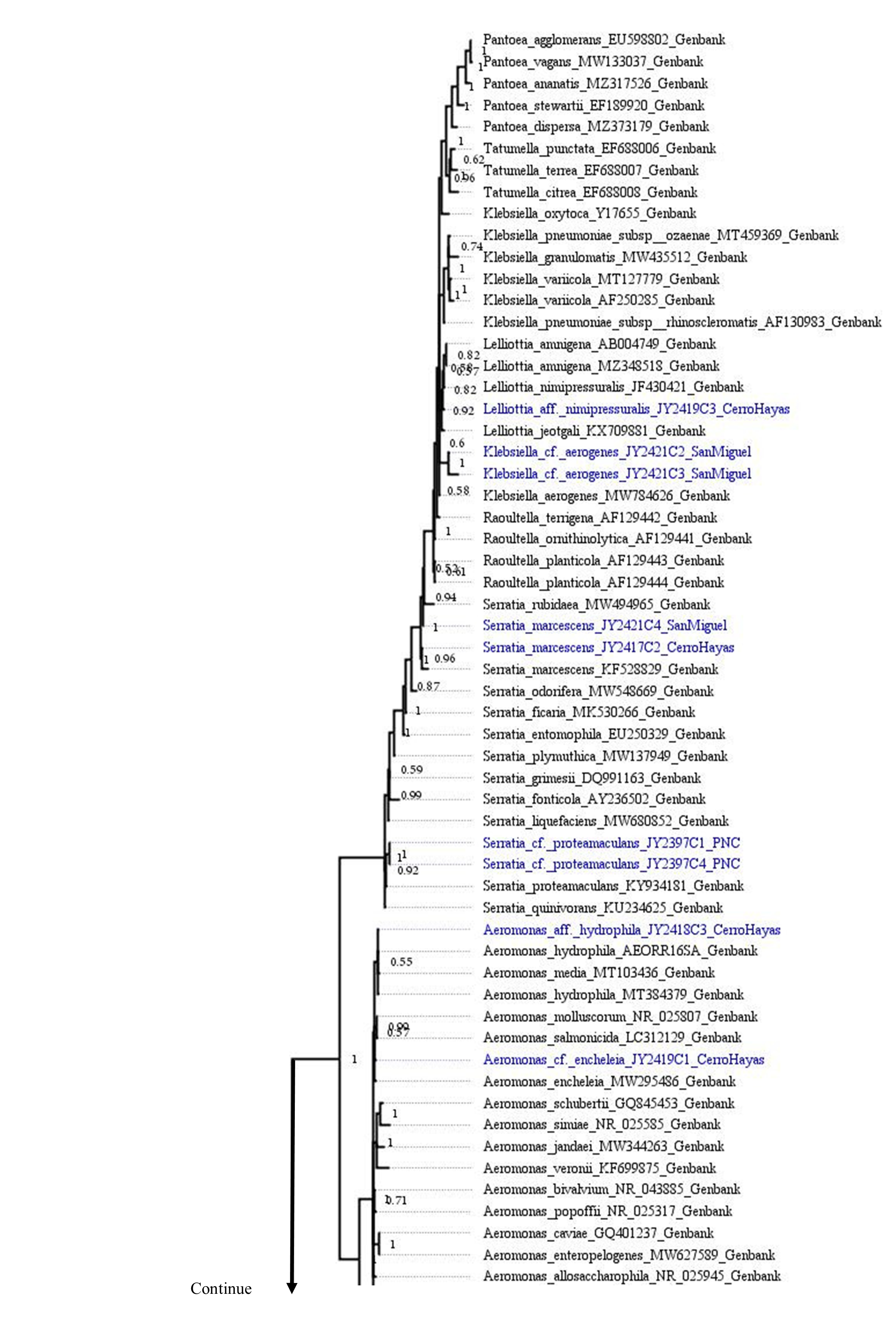

Supplement: Figure S11 — Section (a) of the phylogeny. [file peerj-12-18317-s001.png]

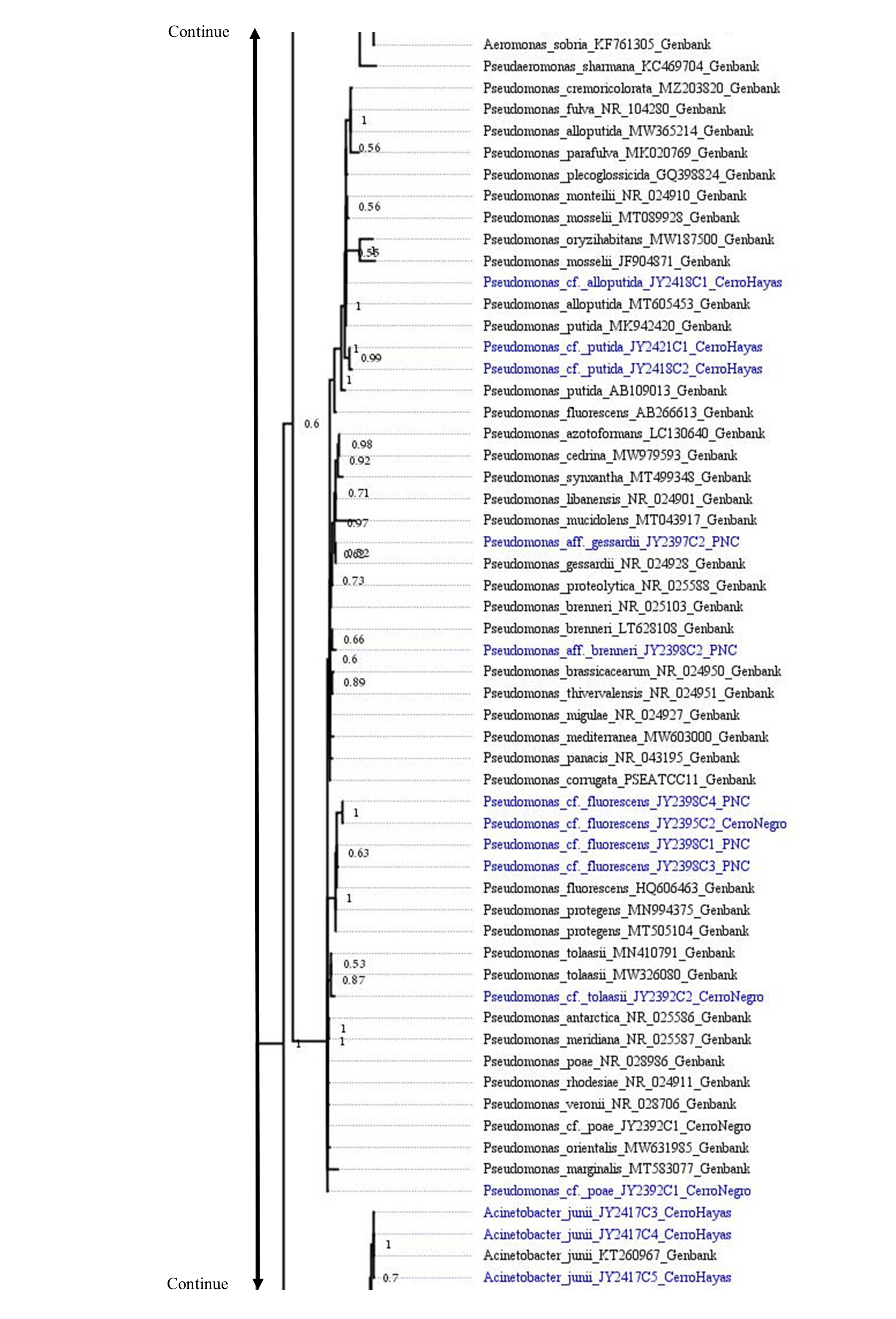

Supplement: Figure S12 — Section (b) of the phylogeny [file peerj-12-18317-s002.png]

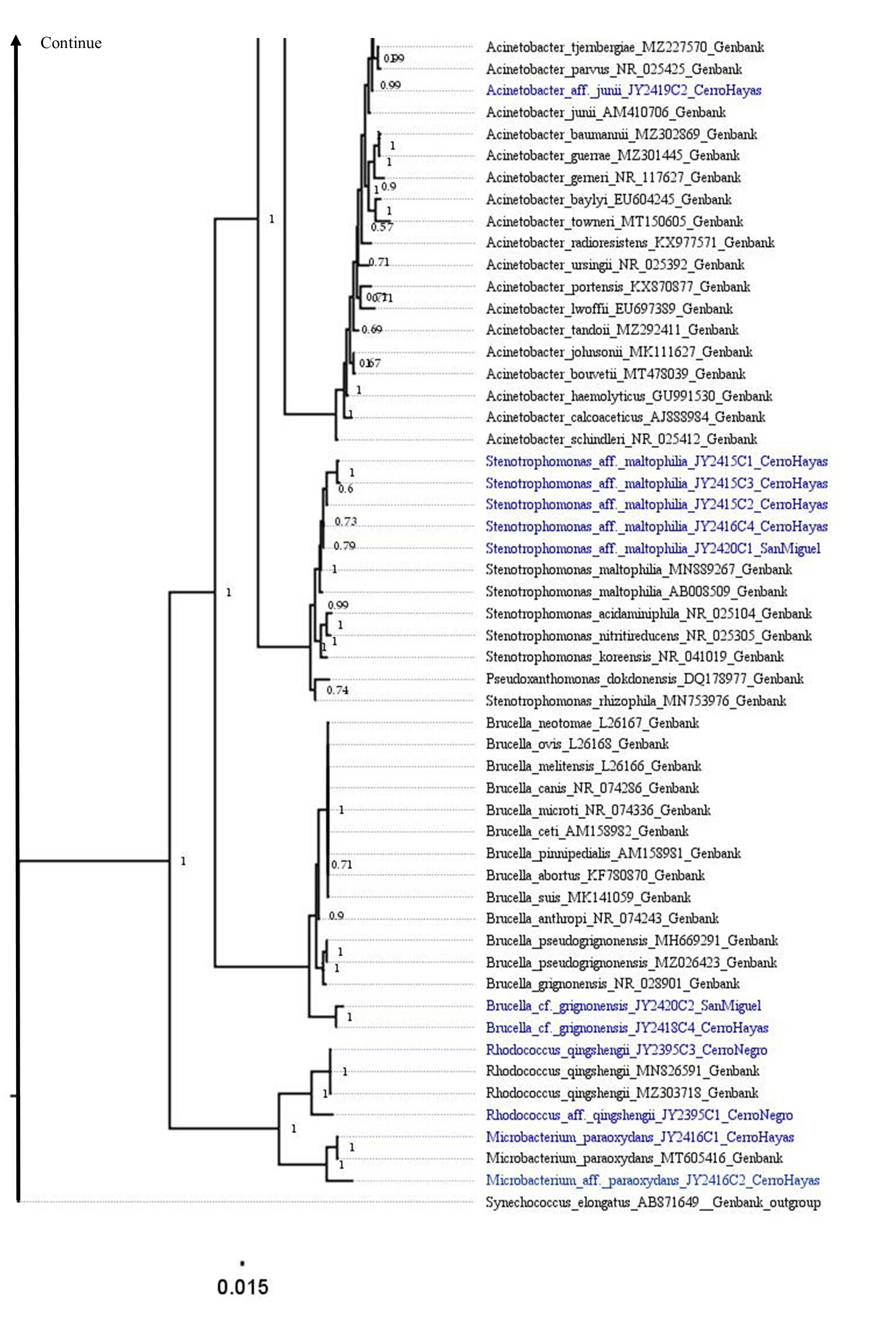

Supplement: Figure S13 — Section (c) of the phylogeny [file peerj-12-18317-s003.png]
