# Supplementary material for: Molecular detection of Batrachochytrium dendrobatidis (Chytridiomycota) and culturable skin bacteria associated with three critically endangered species of Atelopus (Anura: Bufonidae) in Ecuador
Source: PeerJ. 2024 Oct 24;12:e18317. doi: 10.7717/peerj.18317 (PMC11512805; doi:10.7717/peerj.18317)
Supplement: Table S1 — Data on isolated strains, taxonomic classification and maximum identity from BLASTn [file peerj-12-18317-s004.docx]

**Table S1:** **Preliminary information of the bacterial isolates was given by BLASTn analysis.**

| **Isolate code** | **Phylum** | **Class, Order** | **Family** | **Genus** | **Probable species** | **Maximun % Identity** |
| --- | --- | --- | --- | --- | --- | --- |
| JY2395C1 | Actinobacteria | Actinobacteria, Actinomycetales | Corynebacteriaceae | *Rhodococcus* | *R. qingshengii* | 100 |
| JY2395C3 |  |  |  |  |  | 100 |
| JY2416C1 |  |  | Microbacteriaceae | *Microbacterium* | *M. paraoxydans* | 99.80 |
| JY2416C2 |  |  |  |  |  | 100 |
| JY2418C4 | Proteobacteria | Alphaproteobacteria, Rhizobiales | Brucellaceae | *Brucella* | *B. anthropi* | 99.90 |
| JY2420C2 |  |  |  |  | *B. pseudogrignonensis* | 99.90 |
| JY2418C3 |  | Gammaproteobacteria, Aeromonadales | Aeromonadaceae | *Aeromonas* | *A. hydrophila* | 100 |
| JY2419C1 |  |  |  |  | *A. encheleia* | 99.80 |
| JY2417C2 |  | Gammaproteobacteria, Enterobacterales | Yersiniaceae | *Serratia* | *S. marcescens* | 99.90 |
| JY2421C4 |  |  |  |  |  | 100 |
| JY2397C1 |  |  |  |  | *S. proteamaculans* | 99.60 |
| JY2397C4 |  |  |  |  |  | 99.59 |
| JY2419C3 |  |  | Enterobacteriaceae | *Lelliottia* | *L. amnigena* | 99.70 |
| JY2421C2 |  |  |  | *Klebsiella* | *K. aerogenes* | 99.60 |
| JY2421C3 |  |  | Enterobacterias | *Pantoea* | *P. agglomerans* | 99.80 |
| JY2417C3 |  | Gammaproteobacteria, Pseudomonadales | Moraxellaceae | *Acinetobacter* | *A. junii* | 99.90 |
| JY2417C4 |  |  |  |  |  | 99.90 |
| JY2417C5 |  |  |  |  |  | 99.80 |
| JY2419C2 |  |  |  |  | *A. calcoaceticus* | 99.80 |
| JY2418C1 |  |  | Pseudomonadaceae | *Pseudomonas* | *P. alloputida* | 99.80 |
| JY2421C1 |  |  |  |  |  | 100 |
| JY2398C2 |  |  |  |  | *P. brenneri* | 99.90 |
| JY2418C2 |  |  |  |  | *P. mosselii* | 99.90 |
| JY2397C2 |  |  |  |  | *P. fluorescens* | 100 |
| JY2398C1 |  |  |  |  |  | 99.80 |
| JY2398C3 |  |  |  |  |  | 99.70 |
| JY2398C4 |  |  |  |  |  | 99.80 |
| JY2392C1 |  |  |  |  | *P. poae* | 99.41 |
| JY2395C2 |  |  |  |  | *P. protegens* | 100 |
| JY2392C2 |  |  |  |  | *P. tolaasii* | 99.80 |
| JY2415C1 |  | Gammaproteobacteria, Xanthomonadales | Xanthomonadaceae | *Stenotrophomonas* | *S. maltophilia* | 99.80 |
| JY2415C2 |  |  |  |  |  | 99.70 |
| JY2415C3 |  |  |  |  |  | 99.41 |
| JY2416C4 |  |  |  |  |  | 99.80 |
| JY2420C1 |  |  |  |  |  | 100 |
