# Supplementary material for: Molecular detection of Batrachochytrium dendrobatidis (Chytridiomycota) and culturable skin bacteria associated with three critically endangered species of Atelopus (Anura: Bufonidae) in Ecuador
Source: PeerJ. 2024 Oct 24;12:e18317. doi: 10.7717/peerj.18317 (PMC11512805; doi:10.7717/peerj.18317)
Supplement: Table S2 — Isolated code, probable species, scores and accession Genbank accession numbers [file peerj-12-18317-s005.docx]

**Table S2: Summarized similarity measures of the identified bacterial species using 16S rRNA gene by BLASTn analysis.**

| **Isolate code** | **Probable species** | **Maximum score** | **Total score** | **% Query cover** | **E- value** | **Maximum % Identity** | **GenBank Accession Number** |
| --- | --- | --- | --- | --- | --- | --- | --- |
| JY2395C1 | *R. qingshengii* | 828 | 828 | 100 | 0 | 100 | [MN826591.1](https://www.ncbi.nlm.nih.gov/nucleotide/MN826591.1?report=genbank&log$=nucltop&blast_rank=2&RID=C4SH2ND801R) |
| JY2395C3 |  | 1783 | 1783 | 100 | 0 | 100 | [MN826591.1](https://www.ncbi.nlm.nih.gov/nucleotide/MN826591.1?report=genbank&log$=nucltop&blast_rank=2&RID=C4RAZYT2013) |
| JY2416C1 | *M. paraoxydans* | 1829 | 1829 | 100 | 0 | 99.80 | [MT605416.1](https://www.ncbi.nlm.nih.gov/nucleotide/MT605416.1?report=genbank&log$=nucltop&blast_rank=1&RID=C5A97KVP013) |
| JY2416C2 |  | 1840 | 1840 | 100 | 0 | 100 | [MT605416.1](https://www.ncbi.nlm.nih.gov/nucleotide/MT605416.1?report=genbank&log$=nucltop&blast_rank=1&RID=C5A97KVP013) |
| JY2418C4 | *B. anthropi* | 1792 | 1792 | 99 | 0 | 99.90 | [MT534544.1](https://www.ncbi.nlm.nih.gov/nucleotide/MT534544.1?report=genbank&log$=nucltop&blast_rank=1&RID=KCN25VDZ016) |
| JY2420C2 | *B. pseudogrignonensis* | 1818 | 1818 | 99 | 0 | 99.90 | [MH669291.1](https://www.ncbi.nlm.nih.gov/nucleotide/MH669291.1?report=genbank&log$=nucltop&blast_rank=1&RID=C53RHA03013) |
| JY2418C3 | *A. hydrophila* | 1845 | 1845 | 99 | 0 | 100 | [MT384379.1](https://www.ncbi.nlm.nih.gov/nucleotide/MT384379.1?report=genbank&log$=nucltop&blast_rank=2&RID=C580W4UF013) |
| JY2419C1 | *A. encheleia* | 1864 | 1864 | 100 | 0 | 99.80 | [MN999980.1](https://www.ncbi.nlm.nih.gov/nucleotide/MN999980.1?report=genbank&log$=nucltop&blast_rank=3&RID=C57HRKRY013) |
| JY2417C2 | *S. marcescens* | 1858 | 1858 | 99 | 0 | 99.90 | [MT538443.1](https://www.ncbi.nlm.nih.gov/nucleotide/MT538443.1?report=genbank&log$=nucltop&blast_rank=1&RID=C59ZG0KW016) |
| JY2421C4 |  | 1803 | 1803 | 100 | 0 | 99.60 | MK530287.1 |
| JY2397C1 | *S. proteamaculans* | 1783 | 1783 | 100 | 0 | 99.59 | [MK530287.1](https://www.ncbi.nlm.nih.gov/nucleotide/MK530287.1?report=genbank&log$=nucltop&blast_rank=1&RID=KDE0AYXU013) |
| JY2397C4 |  | 1796 | 1796 | 100 | 0 | 100 | [MT101739.1](https://www.ncbi.nlm.nih.gov/nucleotide/MT101739.1?report=genbank&log$=nucltop&blast_rank=2&RID=KDD3T01Z013) |
| JY2419C3 | *L. amnigena* | 1818 | 1818 | 100 | 0 | 99.70 | [MH669129.1](https://www.ncbi.nlm.nih.gov/nucleotide/MH669129.1?report=genbank&log$=nucltop&blast_rank=1&RID=KDE5TEPG013) |
| JY2421C2 | *K. aerogenes* | 1799 | 1799 | 100 | 0 | 99.60 | [MN177204.1](https://www.ncbi.nlm.nih.gov/nucleotide/MN177204.1?report=genbank&log$=nucltop&blast_rank=3&RID=KDF8TP09013) |
| JY2421C3 | *P. agglomerans* | 1805 | 1805 | 100 | 0 | 99.80 | [MT367857.1](https://www.ncbi.nlm.nih.gov/nucleotide/MT367857.1?report=genbank&log$=nucltop&blast_rank=1&RID=C4TV5SZ8013) |
| JY2417C3 | *A. junii* | 1866 | 1866 | 100 | 0 | 99.90 | [KT260967.1](https://www.ncbi.nlm.nih.gov/nucleotide/KT260967.1?report=genbank&log$=nucltop&blast_rank=4&RID=C59BHMXA013) |
| JY2417C4 |  | 1866 | 1866 | 100 | 0 | 99.90 | [KT260967.1](https://www.ncbi.nlm.nih.gov/nucleotide/KT260967.1?report=genbank&log$=nucltop&blast_rank=4&RID=C59BHMXA013) |
| JY2417C5 |  | 1862 | 1862 | 99 | 0 | 99.80 | [KT260967.1](https://www.ncbi.nlm.nih.gov/nucleotide/KT260967.1?report=genbank&log$=nucltop&blast_rank=4&RID=C590EBEH013) |
| JY2419C2 | *A. calcoaceticus* | 1851 | 1851 | 99 | 0 | 99.80 | [MG011543.1](https://www.ncbi.nlm.nih.gov/nucleotide/MG011543.1?report=genbank&log$=nucltop&blast_rank=1&RID=C56CUY3K01R) |
| JY2418C1 | *P. alloputida* | 1853 | 1853 | 100 | 0 | 99.80 | [MT605453.1](https://www.ncbi.nlm.nih.gov/nucleotide/MT605453.1?report=genbank&log$=nucltop&blast_rank=1&RID=C58RMBS4013) |
| JY2421C1 |  | 1851 | 1851 | 100 | 0 | 100 | [MT605453.1](https://www.ncbi.nlm.nih.gov/nucleotide/MT605453.1?report=genbank&log$=nucltop&blast_rank=1&RID=C533Z4KE013) |
| JY2398C2 | *P. brenneri* | 1869 | 1869 | 99 | 0 | 99.90 | [LT628108.1](https://www.ncbi.nlm.nih.gov/nucleotide/LT628108.1?report=genbank&log$=nucltop&blast_rank=1&RID=C5D1RVYR016) |
| JY2418C2 | *P. mosselii* | 1862 | 1862 | 99 | 0 | 99.90 | [MT089928.1](https://www.ncbi.nlm.nih.gov/nucleotide/MT089928.1?report=genbank&log$=nucltop&blast_rank=9&RID=C589JHU9013) |
| JY2397C2 | *P. fluorescens* | 1821 | 1821 | 100 | 0 | 100 | [MN715320.1](https://www.ncbi.nlm.nih.gov/nucleotide/MN715320.1?report=genbank&log$=nucltop&blast_rank=1&RID=KDDPZGP7013) |
| JY2398C1 |  | 1855 | 1855 | 100 | 0 | 99.80 | [HQ606463.1](https://www.ncbi.nlm.nih.gov/nucleotide/HQ606463.1?report=genbank&log$=nucltop&blast_rank=1&RID=C5BW6E68013) |
| JY2398C3 |  | 1858 | 1858 | 100 | 0 | 99.70 | [HQ606463.1](https://www.ncbi.nlm.nih.gov/nucleotide/HQ606463.1?report=genbank&log$=nucltop&blast_rank=1&RID=C5BW6E68013) |
| JY2398C4 |  | 1866 | 1866 | 100 | 0 | 99.80 | [HQ606463.1](https://www.ncbi.nlm.nih.gov/nucleotide/HQ606463.1?report=genbank&log$=nucltop&blast_rank=1&RID=C5BW6E68013) |
| JY2392C1 | *P. poae* | 1230 | 1230 | 100 | 0 | 99.41 | [MT631989.1](https://www.ncbi.nlm.nih.gov/nucleotide/MT631989.1?report=genbank&log$=nucltop&blast_rank=1&RID=C5E8GE4N013) |
| JY2395C2 | *P. protegens* | 1786 | 1786 | 100 | 0 | 100 | [MT505104.1](https://www.ncbi.nlm.nih.gov/nucleotide/MT505104.1?report=genbank&log$=nucltop&blast_rank=1&RID=C4RVPF30016) |
| JY2392C2 | *P. tolaasii* | 1858 | 1858 | 99 | 0 | 99.80 | [MN410791.1](https://www.ncbi.nlm.nih.gov/nucleotide/MN410791.1?report=genbank&log$=nucltop&blast_rank=1&RID=C5753M12013) |
| JY2415C1 | *S. maltophilia* | 1871 | 1871 | 100 | 0 | 99.80 | [MN006523.1](https://www.ncbi.nlm.nih.gov/nucleotide/MN006523.1?report=genbank&log$=nucltop&blast_rank=1&RID=C5B438XE016) |
| JY2415C2 |  | 1868 | 1868 | 99 | 0 | 99.70 | [MT256163.1](https://www.ncbi.nlm.nih.gov/nucleotide/MT256163.1?report=genbank&log$=nucltop&blast_rank=3&RID=C5AVBFUK013) |
| JY2415C3 |  | 1844 | 1844 | 100 | 0 | 99.41 | [MT256163.1](https://www.ncbi.nlm.nih.gov/nucleotide/MT256163.1?report=genbank&log$=nucltop&blast_rank=3&RID=C5AVBFUK013) |
| JY2416C4 |  | 1857 | 1857 | 99 | 0 | 99.80 | [MN006523.1](https://www.ncbi.nlm.nih.gov/nucleotide/MN006523.1?report=genbank&log$=nucltop&blast_rank=1&RID=C5B438XE016) |
| JY2420C1 |  | 1864 | 1864 | 99 | 0 | 100 | [MN889267.1](https://www.ncbi.nlm.nih.gov/nucleotide/MN889267.1?report=genbank&log$=nucltop&blast_rank=3&RID=C54TT8XR013) |
