# Supplementary material for: Molecular detection of Batrachochytrium dendrobatidis (Chytridiomycota) and culturable skin bacteria associated with three critically endangered species of Atelopus (Anura: Bufonidae) in Ecuador
Source: PeerJ. 2024 Oct 24;12:e18317. doi: 10.7717/peerj.18317 (PMC11512805; doi:10.7717/peerj.18317)
Supplement: Table S3 — Distance percentages between the sequences of the isolated strains, and the DNA sequences of the possible species from GenBank. [file peerj-12-18317-s006.docx]

**Table S3: Bacterial identification by phylogenetic analysis, distance percentages between the sequences of the isolates, and the DNA sequences of the possible species from GenBank.**

| **Isolate code** | **Genus** | **Probable species** | **% Distance** | **Clade** |
| --- | --- | --- | --- | --- |
| JY2419C2 | *Acinetobacter* | *Acinetobacter aff. junii* | 98,72 | Clade 1 |
| JY2417C3 |  | *Acinetobacter junii* | 100 | Clade 2 |
| JY2417C4 |  |  | 99,90 |  |
| JY2417C5 |  |  | 99,80 |  |
| JY2418C3 | *Aeromonas* | *Aeromonas aff. hydrophila* | 99,90 | Clade 3 |
| JY2419C1 |  | *Aeromonas cf. encheleia* | 99,61 | Clade 4 |
| JY2418C4 | *Brucella* | *Brucella cf. grignonensis* | 93,77 | Clade 5 |
| JY2420C2 |  |  | 93,51 |  |
| JY2421C2 | *Klebsiella* | *Klebsiella cf. aerogenes* | 97,16 | Clade 6 |
| JY2421C3 |  |  | 95,93 |  |
| JY2419C3 | *Lelliottia* | *Lelliottia aff. nimipressuralis* | 99,63 | Clade 7 |
| JY2416C2 | *Microbacterium* | *Microbacterium aff. paraoxydans* | 93,04 | Clade 8 |
| JY2416C1 |  | *Microbacterium paraoxydans* | 100 | Clade 9 |
| JY2398C2 | *Pseudomonas* | *Pseudomonas aff. brenneri* | 99,01 | Clade 10 |
| JY2397C2 |  | *Pseudomonas aff. gessardii* | 99,92 | Clade 11 |
| JY2418C1 |  | *Pseudomonas cf. alloputida* | 99,90 | Clade 12 |
| JY2395C2 |  | *Pseudomonas cf. fluorescens* | 98,6 | Clade 13 |
| JY2398C1 |  |  | 100 |  |
| JY2398C3 |  |  | 99,70 |  |
| JY2398C4 |  |  | 98,81 |  |
| JY2392C1 |  | *Pseudomonas cf. poae* | 100 | Clade 14 |
| JY2418C2 |  | *Pseudomonas cf. putida* | 98,42 | Clade 15 |
| JY2421C1 |  |  | 99,10 |  |
| JY2392C2 |  | *Pseudomonas cf. tolaasii* | 99,01 | Clade 16 |
| JY2395C1 | *Rhodococcus* | *Rhodococcus aff. qingshengii* | 91,54 | Clade 17 |
| JY2395C3 |  | *Rhodococcus qingshengii* | 100 | Clade 18 |
| JY2421C4 | *Serratia* | *Serratia cf. marcescens* | 98,97 | Clade 19 |
| JY2397C1 |  | *Serratia cf. proteamaculans* | 99,29 | Clade 20 |
| JY2397C4 |  |  | 99,28 |  |
| JY2417C2 |  | *Serratia marcescens* | 97,64 | Clade 21 |
| JY2415C1 | *Stenotrophomonas* | *Stenotrophomonas aff. maltophilia* | 97,13 | Clade 22 |
| JY2415C2 |  |  | 99,12 |  |
| JY2415C3 |  |  | 97,13 |  |
| JY2416C4 |  |  | 99,60 |  |
| JY2420C1 |  |  | 99,90 |  |
